# Revisiting the Hetero-Fertilization Phenomenon in Maize

**DOI:** 10.1371/journal.pone.0016101

**Published:** 2011-01-19

**Authors:** Shibin Gao, Raman Babu, Yanli Lu, Carlos Martinez, Zhuanfang Hao, Alan F. Krivanek, Jiankang Wang, Tingzhao Rong, Jonathan Crouch, Yunbi Xu

**Affiliations:** 1 Maize Research Institute, Sichuan Agricultural University, Ya'an, China; 2 International Maize and Wheat Improvement Center (CIMMYT), Texcoco, México; 3 Institute of Crop Science, National Key Facilities for Crop Genetic Resources and Improvement, Chinese Academy of Agricultural Sciences, Beijing, China; 4 Institute of Crop Science/International Maize and Wheat Improvement Center (CIMMYT), National Key Facilities for Crop Genetic Resources and Improvement, Chinese Academy of Agricultural Sciences, Beijing, China; East Carolina University, United States of America

## Abstract

Development of a seed DNA-based genotyping system for marker-assisted selection (MAS) has provided a novel opportunity for understanding aberrant reproductive phenomena such as hetero-fertilization (HF) by observing the mismatch of endosperm and leaf genotypes in monocot species. In contrast to conventional approaches using specific morphological markers, this approach can be used for any population derived from diverse parental genotypes. A large-scale experiment was implemented using seven F_2_ populations and four three-way cross populations, each with 534 to 1024 individuals. The frequency of HF within these populations ranged from 0.14% to 3.12%, with an average of 1.46%. The highest frequency of HF in both types of population was contributed by the pollen gametes. Using three-way crosses allowed, for the first time, detection of the HF contributed by maternal gametes, albeit at very low frequency (0.14%–0.65%). Four HF events identified from each of two F_2_ populations were tested and confirmed using 1032 single nucleotide polymorphic markers. This analysis indicated that only 50% of polymorphic markers can detect a known HF event, and thus the real HF frequency can be inferred by doubling the estimate obtained from using only one polymorphic marker. As expected, 99% of the HF events can be detected by using seven independent markers in combination. Although seed DNA-based analysis may wrongly predict plant genotypes due to the mismatch of endosperm and leaf DNA caused by HF, the relatively low HF frequencies revealed with diverse germplasm in this study indicates that the effect on the accuracy of MAS is limited. In addition, comparative endosperm and leaf DNA analysis of specific genetic stocks could be useful for revealing the relationships among various aberrant fertilization phenomena including haploidy and apomixis.

## Introduction

The maize seed comprises two major components, the embryo and the endosperm, both originating from the double fertilization process. The embryogenetic process allows the formation of a well-differentiated embryonic axis, surrounded by a single massive cotyledon, the scutellum. At maturity the embryo axis comprises all tissues that will give rise to the seedling structure; root and shoot primordia located at opposite poles and a stem with five or six internodes bearing a leaf at each node (for a detailed description of maize seed anatomy, see [1]). The double fertilization event which is observed throughout the angiosperms is unique among living organisms, and is considered a fundamental component of the evolutionary success of angiosperms [2]. The endosperm is a highly specialized tissue with nutritive function which maximizes the germination success of seeds. Embryo and endosperm are genetically identical, except for ploidy level, with a 2∶1 ratio of maternal to paternal genomes.

**Figure 1:**
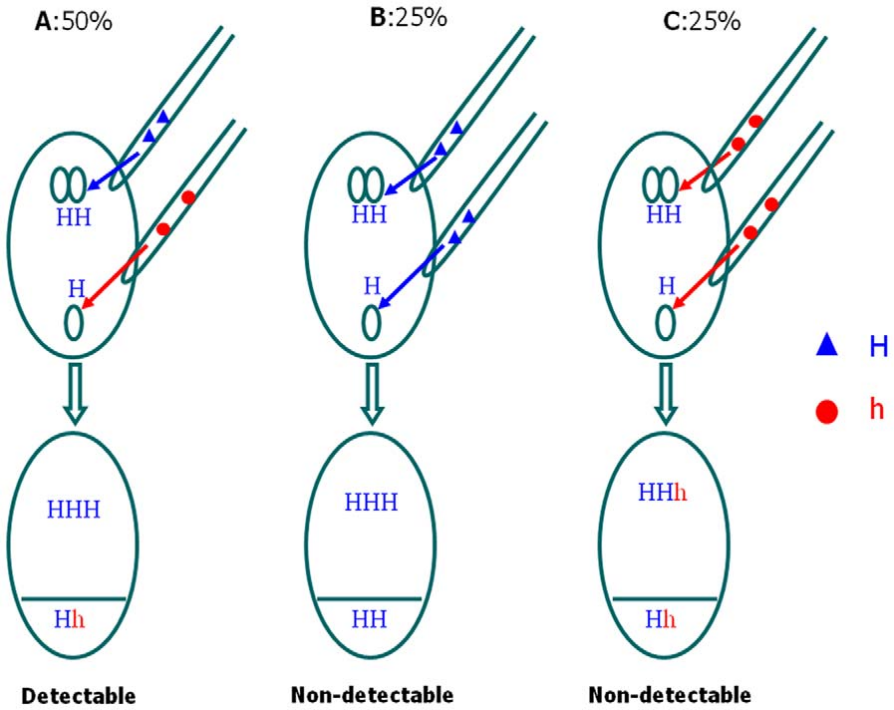
A diagram showing the probability for an HF event to be detected by using one polymorphic marker. For a given HF event and a given segregating marker locus within a population, two independent sperm cells derived from two different pollen grains, represented by H (triangle) and h (circle), respectively, can be only detected, with the probability of 50% (A), when the two pollen grains carry different alleles. However, when the two pollen grains carry the same allele (either H or h), the HF event is not detectable. The chance for the two pollen grains to carry the same H (B) or h (C) is 25%.

Maize normally produces tricellular pollen with one vegetative nucleus and two genetically identical sperm cells [3]. As a defining feature of the angiosperms, the double-fertilization involves a process, in which one of the sperm (*n*) fertilizes the egg cell (*n*) in an ovule to form an embryo (*n*+*n* = 2*n*) and the other sperm cell (*n*) fertilizes the central cell/polar nuclei (2*n*) to form the endosperm (2*n*+*n* = 3*n*) [2], [4]–[6]. In maize, an aberrant mode of fertilization called hetero-fertilization (HF) occurs when the egg cell (*n*) and the central cell (or polar nuclei 2*n*) of the same ovule are fertilized by genetically non-identical sperm cells released from different pollen grains (Figure 1A), or conversely, when egg and polar nuclei are of different genetic constitution and fuse with identical sperms. The HF phenomenon has been investigated by several researchers using morphological markers [7]–[10]. Previous studies have estimated the incidence of HF in maize to be on an average 1.25% [8], although significant variation was observed in different germplasm. A later study by Robertson [10] reported up to 5% HF in diverse germplasm of maize. Sprague [8] reported a rare genotype of maize in which 25% HF frequency was observed. A recent report [6] studied HF rates of trifluralin induced bicellular pollen, and reported 3.7–4.8% HF in 0.3% trefanocide solution on diploid-diploid crosses, while the control treatment exhibited significantly lower HF rates (2.3%). When studying tetraploid-diploid crosses, both 0.3% trefanocide treatment as well as control produced HF kernels in fairly high frequency, ranging from 33 to 48%. More recently, an interesting experiment [11] using a dual pollination method identified single fertilization events in seven maize lines across different genetic backgrounds, in which fertilization of egg cells occurred even though central cells were not fertilized, and suggested that at least one-fifth of HF events could be the result of single fertilization events in maize.

In all such classical approaches, HF could only be studied using genes that control color production in the aleurone and scutellum, which are expressed as externally observable phenotypes. In crosses with male parents that were heterozygous for one or more of the color genes and female parents that were recessive tester lines, HF is most easily recognized as seed with colorless aleurone and colored scutellum. On the ears on plants resulting from such seeds, there is a reciprocal HF class that will have colored aleurone and colorless scutellum [10]. However, exclusive dependence on morphological markers such as color may lead to inaccurate estimates of HF frequency. This is because the underlying anthocyanin and carotenoid synthesis pathways in maize are typically influenced by a number of environment effects and dosage sensitive genes [12]. Similar accuracy problems are reported for identification of haploids using pigmentation markers [13]. On the other hand, use of morphological markers cannot identify the HF events resulting from maternal gametes.

The phenomenon of HF has implications for the accuracy of seed DNA-based genotyping, which involves excising a portion of the endosperm to generate a source of tissue for subsequent DNA extraction [14]. Endosperm (seed) – embryo (plant) genotype mismatches could seriously affect the accuracy of seed-DNA based genotyping if the rate of HF was sufficiently high. On the other hand, such a nondestructive seed-sampling method that allows germination of the sampled seed and permits selections to be carried out in advance of planting which could lead to significant savings of field space and cost. This will in turn enable scientists to work with substantially larger effective population sizes for mapping complex agronomic traits using selective phenotyping approaches [15]. In addition, this genotyping system has been found to be a valuable technique for rapidly validating marker-trait associations especially for kernel quality traits such as provitaimn-A and high lysine and/or tryptophan in maize, where individual seeds of a segregating population are grouped based on the genotype and used in biochemical phenotyping without having to grow them out in the field (CIMMYT unpublished results). Genomic DNA sequence polymorphisms are abundant and are not influenced by the environment or diverse genetic backgrounds. Thus, DNA markers in combination with our seed DNA-based genotyping system allows DNA extraction from the endosperm as well as the embryo of single seeds, and provides an unprecedented opportunity to study aberrant reproductive phenomena including the HF phenomenon in detail.

The major objectives of this investigation were to generate reliable estimates of HF frequency across diverse maize lines using molecular markers in order to: a) standardize a method of estimating naturally occurring HF frequency using molecular markers; (b) determine the rate and extent of HF across different sources of maize germplasm that are routinely used in various breeding programs; and (c) obtain experimental evidences to verify through controlled reciprocal three-way crosses whether HF can be caused by maternal factors in addition to pollen. As HF causes the embryo genotype of a given maize kernel to be significantly different from its corresponding endosperm genotype, accurate estimation of HF will help us evaluate the potential risk of erroneous results during seed DNA-based marker-assisted selection (MAS).

## Results

### HF frequencies in seven F_2_ populations

**Figure 2:**
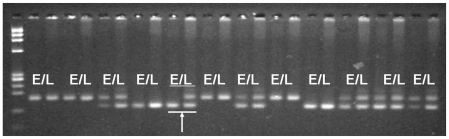
Hetero-fertilization event detected in F_2_ populations. Genotype difference between endosperm DNA (E) and embryo DNA (L) can be revealed by one polymorphic SSR marker as shown by the arrow.

**Figure 3:**
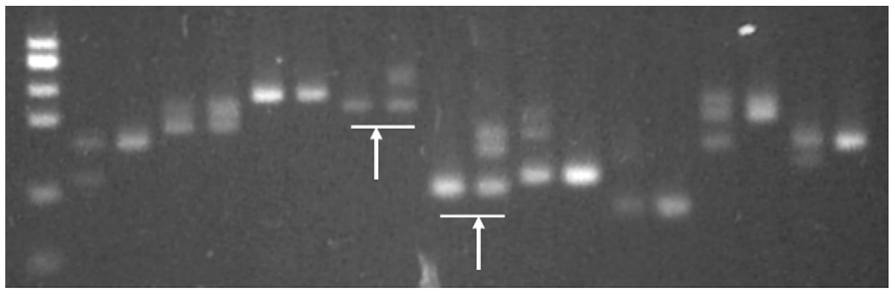
Confirmation of hetero-fertilization events. Nine polymorphic SSRs were used to confirm hetero-fertilization events detected in F_2_ populations. After completely excluding the interference from residual pericarp, once one SSR, as shown by arrows, is identified to amplify a homozygous endosperm (the underlined left lane) but a heterozygous leaf (the underline right lane), the HF event is confirmed.

For detection of HF frequencies, we screened five of the F_2_ populations (Table 1) with a single SSR marker that was clearly polymorphic between the parental lines and easily scorable in segregating F_2_ individuals using simple agarose gel systems (Figure 2). The two other F_2_ populations (Table 1) were segregating for two important genes in the carotenoid synthetic pathway (LycE and HydB), so we used STS markers for these two genes instead. The HF frequencies estimated through this analysis are shown in Table 2. Hetero-fertilized seeds were observed in all seven F_2_ populations, including those with a homozygous endosperm and a heterozygous embryo (AAA/AB or BBB/AB) as well as those with a heterozygous endosperm and a homozygous embryo (AAB/AA or BBA/BB). To avoid false HF identifications caused by pericarp contamination (as opposed to a heterozygous endosperm and homozygous embryo), up to ten polymorphic SSR markers at other loci were screened in search of a homozygous endosperm with a heterozygous embryo (Figure 3). In contrast, detection of a homozygous endosperm with a heterozygous embryo is unambiguous because the pericarp is always derived from maternal tissue and exhibits a heterozygous genotype for all polymorphic markers. Based on the single marker analysis, the frequency of HF in the seven F_2_ populations ranged from 1.02% to 3.12% (Table 2). For two F_2_ populations, HP6 and HP7, the two STS markers revealed different HF frequencies for the same population as big as those observed among the five F2 populations.

**Table 1:** Segregating populations derived from diverse maize germplasm used for hetero-fertilization detection in this study.

Name	Cross model	Germplasm source and description of kernel characteristics
*Five F_2_ populations*		
HP1	Line 1 × Line 2	Line 1: Progeny of P390bcoC3F191 and P73TLC3, white-flint kernel
		Line 2: Progeny of LPSEQC7, white-flint kernel
HP2	Line 3 × Line 4	Line 3: Progeny of CL-RCW46, white-dent kernel
		Line 4: Progeny of CL-RCW84, white-flint kernel
HP3	Line 5 × Line 6	Line 5: Progeny of MIRC5, white-dent kernel
		Line 6: Progeny of CL-RCW45, white-flint kernel
HP4	Line 7 × Line 8	Line 7: Progeny of CML464 and CML175, yellow-flint kernel
		Line 8: Progeny of CML176 and BTZTVC PR93A, yellow-flint kernel
HP5	CML492×CML494	Public inbred lines released by CIMMYT
HP6	Line 9 X Line 10	Line 9: KUI carotenoid syn-FS11-1-1-B-B-BLine10: KU1409/DE3/KU1409)S2-18-2-B)-B
HP7	Line 11 X Line 10	Line11: CML297
*Four three-way crosses*		
HP8	(Line 3×Line 4) ×CML494	Described as above
HP9	(CML484×CML312) ×CML494	Described as above
HP10	CML461×(Line 7×Line 8)	Described as above
HP11	CML246×(Line 7×Line 8)	CML246, highland adaptation, white and semi-dent kernel

**Table 2:** Hetero-fertilization frequencies as detected by one or two markers in seven F_2_ populations.

Population (A×B)	SSR/STS	Number of normal cases (E/L)			Number of hetero-fertilization events (E/L)		Total	Ratio (%)
		AAA/AA	BBB/BB	AAB/AB	AAA/AB +BBB/AB	AAB/AA+BBA/BB		
HP1	Umc1040	142	123	263	1+4	1+0	534	1.12
HP2	Umc1015	94	340	438	2+2	2+3	881	1.02
HP3	Bnlg1270	171	191	311	4+4	4+3	688	2.18
HP4	Umc1008	185	144	332	4+2	2+0	669	1.19
HP5	Umc1071	292	246	468	3+9	1+5	1024	1.76
HP6	LycE5′TE	26	202	125	3+2	4+2	353	3.12
	HydB3′TE	79	91	183	1+2	3+2	353	2.27
HP7	LycE5′TE	58	217	101	1+4	1+2	376	2.13
	HydB3′TE	93	80	203	0+2	1+1	376	1.06

Note: The pollination model for five F_2_ populations is A×B, where A is used as female parent and B as male parent. For consistency, the genotypes of SSR allele combination in endosperm (E) and leaf (L) are shown using the same symbol with the pollination model A and B.

### HF phenomenon in four three-way cross populations

**Figure 4:**
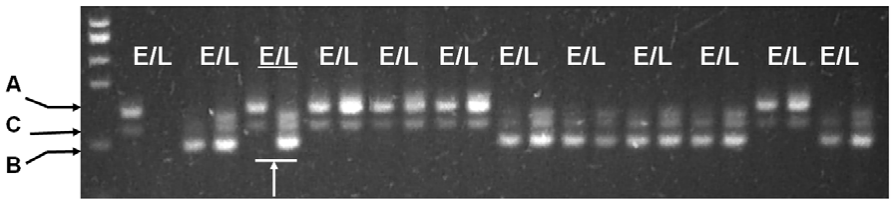
Detection of hetero-fertilization event contributed by maternal gametophyte in three-way cross populations. An HF plant in HP9 was detected as shown by the arrow. E: endosperm, L: leaf; A, B and C: three alleles derived from three different parents in the cross model (A×B)×C.

**Figure 5:**
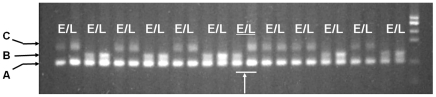
Detection of hetero-fertilization event contributed by different pollen grains in three-way cross populations. An HF plant in HP10 was detected as shown by the arrow. E: endosperm, L: leaf; A, B and C: three alleles derived from three different parents in the cross model (A×B)×C.

Using F_2_ segregating population, we can investigate the general rate of HF occurring in ordinary germplasm but we cannot determine which parental genotype contributes to the HF. Therefore, three-way cross populations were generated in order to study this component (Table 1). Seeds from the two populations (HP8 and HP9), generated from three-way crosses were harvested from the crossing model (A×B)×C in which a single cross hybrid was used as the maternal parent and an inbred line was used as the paternal pollen parent. Since the pollen was homozygous, any HF could only have resulted from the maternal gametes. Five HF events (0.65%) were observed from 765 pairwise comparisons in HP8 but only one HF event (0.14%) was observed from 716 pairwise comparisons in HP9 (Table 3; Figure 4). To our knowledge, this is the first empirical evidence in maize that HF could be caused by certain abnormal events during the formation of the female gametophyte resulting in cells within the embryo sac with different genetic constitutions. The other two populations generated from three-way crosses (HP10 and HP11), were generated through the crossing model A×(B×C), in which an inbred line was used as maternal parent and single cross hybrids were used as the pollen donor. Eight HF events were identified in HP10 (from 731 pairwise comparisons, Figure 5) and 12 HF events were identified in HP11 (from 877 pairwise comparisons). The frequency of HF in HP10 and HP11 was 1.01% and 1.37%, respectively (Table 3). In these two populations, the HF events could only have resulted from the contribution of genetically non-identical sperm cells from two different pollen tubes during the double fertilization process.

**Table 3:** Hetero-fertilization frequencies detected in four three-way cross populations.

Population	SSR	Number of normal cases (E/L)		Number of hetero- fertilization events (E/L)	Total	Ratio (%)
*(A*×*B)*×*C*		*AAC/AC*	*BBC/BC*	*AAC/BC + BBC/AC*		
HP8	Umc1015	395	365	2+3	765	0.65
HP9	Bnlg1144	369	346	1+0	716	0.14
*A*×*(B*×*C)*		*AAB/AB*	*AAC/AC*	*AAB/AC+ AAC/AB*		
HP10	Umc1805	416	307	2+6	731	1.01
HP11	Bnlg1043	433	432	3+9	877	1.37

Note: The two pollination models for three-way crosses are (A×B)×C and A×(B×C). For consistency, the genotypes of SSR allele combination in endosperm (E) and leaf (L) are shown using the same symbol with the pollination model A, B and C.

### Probability of detecting HF events with different numbers of markers

A total of eight HF events from two F_2_ populations were tested and confirmed using chip-based single nucleotide polymorphism (SNP) genotyping (Table 4). The total number of markers that were used for testing each HF event ranged from 878 to 1028. The numbers of markers that confirmed the HF events, based on the results for endosperm and leaf genotypes not matching, ranged from 127 to 190. Thus, the percentage of all scored markers that identified HF events ranged from 13.2% to 21.6%, with an average of 17.7%, which included all the markers that were monomorphic between the parental lines. As unfortunately we did not include the parental lines for genotyping with the segregating populations, an expected polymorphism rate could be inferred for the tested populations from large-scale genotyping trials that have been done at CIMMYT using the same SNP chip. Based on the average SNP polymorphism rate in maize of 36.3% [16] obtained with 154 diverse maize lines, the percentage of expected polymorphic markers that identified HF events ranged from 36.3% to 59.6%, with an average of 48.9% (Table 4). This indicates that as expected (Figure 1A), there is around a 50% chance that any polymorphic marker will detect an HF event based on the difference between endosperm and leaf genotypes. In other words, one polymorphic marker can only identify 50% of the HF events present in a segregating population. Thus, the real HF frequency can be inferred by doubling the frequency estimated by a single molecular marker that is polymorphic between the parental lines. To directly identify 99% of the HF events in a specific population, at least seven unlinked markers should be used simultaneously as in theory each additional marker can only identify 50% of the HF events that cannot be identified by all previous markers together. As a panel of seven markers can be analyzed in a single multiplex, the cost for genotyping seven markers may not be very much different from the cost for a one-marker analysis. As a result, 99% of erroneous selection events due to the HF events in a MAS breeding program can be eliminated by using seven markers simultaneously.

**Table 4:** Probability of a marker confirming a hetero-fertility event.

Population	Hetero-fertilization event	Total number of markers tested	Number of markers confirming the HF	Probability of detecting HF for all markers (%)	Number of expected polymorphic markers*	Probability of detecting HF for expected polymorphic markers (%)
HP2	1	878	190	21.64	318.7	59.61
	2	980	176	17.96	355.7	49.47
	3	1011	181	17.90	367.0	49.32
	4	902	188	20.84	327.4	57.42
HP3	1	965	127	13.16	350.3	36.26
	2	1028	172	16.73	373.2	46.09
	3	1026	182	17.74	372.4	48.87
	4	997	165	16.55	361.9	45.59
Average				17.73		48.86

*The number of markers that are expected to be polymorphic was estimated using the averaged polymorphism rate (36.3%) as revealed in [16] using the same SNP chip for genotyping.

## Discussion

Due to confounding factors associated with using morphological markers such as the color of different components of the kernel, it is hard to design an experiment to test Sprague's original proposal [8] on paternal and maternal contribution to the HF events. By using molecular marker analysis of three-way crosses, it is now possible to precisely test Sprague's hypothesis. In so doing, we have found that HF events caused by maternal gametes could be detected using the three-way crosses with a single cross as the maternal parent.

Although HF is a rare phenomenon in maize, it may still affect the accuracy of endosperm DNA-based MAS when embryo and endosperm genotypes differ. In some maize genetic stocks, Sprague [8] found HF to be as high as 25%. At this level, the accuracy of seed DNA-based genotyping would be unacceptably compromised. We surveyed 11 populations covering a wide diversity of maize germplasm and observed an average HF frequency of 1.76% in seven F_2_ populations but a relatively lower frequency in three-way crosses which might be due to the fact that two of the three-way crosses were only effective for detecting the HF events contributed by maternal gametes. Our general conclusion is consistent with previous reports, suggesting that there was little variation in frequency of HF incidence in most cultivated maize germplasm [6], [9], [10], [17]. Thus, it seems that the high HF frequency of 25% in the stock tested by Sprague is a very rare exception albeit a useful resource for the study of HF. At a HF frequency of 1–2%, the resultant increase in genotyping errors is acceptable for most MAS applications, particularly if we consider the advantages provided by seed DNA-based genotyping [14].

**Figure 6:**
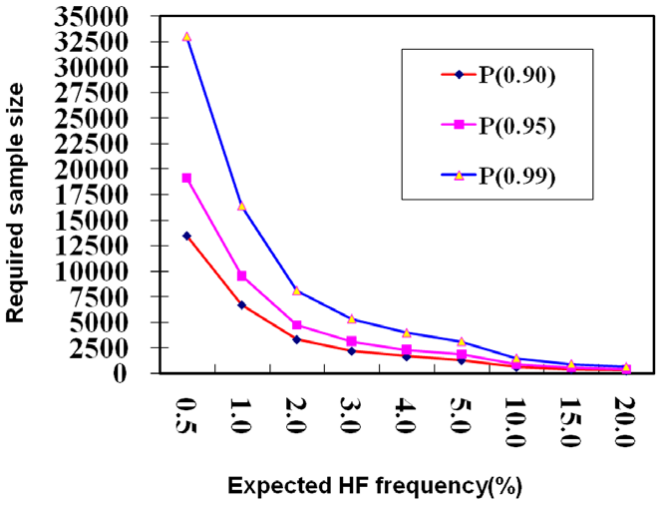
Theoretical expectation for population sizes required for detection of HF events at various expected HF frequencies. Sample sizes required were calculated for each expected HF frequency to ensure to detect HF events at probability levels of 0.90, 0.95 and 0.99, respectively.

Theoretically, a large number of samples are required to accurately estimate the HF frequency due to its low value. The sample size (*n*) required to ensure accurate estimation of a given frequency (*q*) within the confidence interval [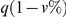,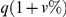] at the probability level 1-α can be calculated by 
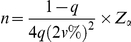
, where 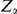 is the inverse of the standard normal cumulative distribution, i.e. 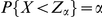 where *X* follows the standard normal distribution. The lower the frequency, the larger the sample size required to generate estimates with a certain level of probability as shown in Figure 6. Based on the HF of 1% to 5% observed in most natural germplasm, 1285 to 6696 samples will be required to ensure an estimation at the 90% significance level. Obviously, such sample sizes are hard to achieve in experimental populations. For example, the sample sizes used in this study ranged from 353 to 1024, with an average of 692, which would provide an estimation of HF frequencies of 1% to 9% at probabilities of 50% to 90%, respectively. Thus, substantially larger sample sizes are required for a highly statistically significant estimation of the HF frequency reported in this study (1.46%). However, the use of 11 segregating populations, all providing similar estimations of HF frequency, provides a substantial level of confidence in the results, as the accumulative probability of getting a right estimation will be equal to the probability in one big population with the population size equal to the sum of sizes for individual populations. In this study, the accumulated population size across the 11 populations is in the region required for detection of HF of 1.46% at the 90% level of confidence.

Kato [18] considered that the aberrant fertilization mechanisms underlying haploidy and HF in maize may be associated. Current explanations suggest that haploids are produced when a single haploid sperm from the pollen grain fertilizes its polar nucleus and the unfertilized egg cell develops parthenogenetically into a haploid plant. However, we did not detect any haploids in this study as there were no haploid inducer lines included in the parental genotypes. Clearly, it would be most interesting to study the HF frequency in double haploid breeding programs and to test whether Sprague's special stock has any haploid inducing tendencies.

Differences in genetic constitution between embryo and endosperm may be common characteristics in haploidy, apomixes and HF. Thus, the genotyping of single mature seeds from carefully chosen or designed segregating populations offers a valuable strategy for basic research of these phenomena in addition to its diverse applications in molecular breeding.

## Materials and Methods

### Plant materials

In this study, maize seeds from two types of hybrid populations were used for DNA extraction and marker analysis: biparental crosses and three-way crosses. For populations derived from biparental crosses, DNA extraction was carried out on F_2_ seeds harvested from the F_1_ plants. Two different three-way crosses were used, A×(B×C) and (A×B) ×C. In the former case, DNA was extracted using the seed harvested from the inbred plants that were pollinated by F_1_ plants, while in the latter case the DNA was extracted from the F_1_ plants that were pollinated with the inbred. The detailed pedigree of parental lines and the corresponding pollination model for the seven biparental F_2_ populations and the four three-way cross populations used in this study are described in Table 1.

### DNA extraction and genotyping

Single seed-based sample collection and DNA extraction using an excised portion of endosperm were performed as described in [14]. Sampled endosperm was transferred into individual 1.1 ml tubes in a 96-tube plate (12 rows each with eight linked tubes, Neptune, CA, USA). The remnant seed was placed in 48-well plates pending DNA analysis results. Leaf tissue of individual plants was collected at 3-leaf stage and the resultant DNA used to represent the embryo genotype in each comparative experiment was extracted using a DNA isolation protocol developed for leaf tissue at CIMMYT [19].

To avoid error and improve efficiency, all operations including endosperm sampling, planting of cutting seed, collection of leaf tissue, and DNA extraction and PCR amplification for both endosperm and leaf samples were performed using plates comprising 12 rows of 8 tubes as the basic unit. When a cutting seed fails to germinate, its corresponding position for the leaf sample will remain empty in the basic unit through collection of leaf DNA to genotyping process, for the convenience of endosperm-leaf sample match. In general, approximately 95% of the sampled seeds germinated for each population.

### Detection of HF events based on polymorphic SSR markers

For both F_2_ and three-way cross populations, HF events were revealed based on the detection of a different genetic constitution between endosperm and embryo (represented by leaf) using one SSR marker that has been screened in advance to show polymorphism among the parental genotypes. In theory, one polymorphic SSR marker can detect half of HF events for both F_2_ and three-way cross populations (as shown in Figure 1). When HF occurs, there is a 50% chance that two sperms have different alleles at a given locus which lead to different genotypes between endosperm and embryo. In contrast, there is also a 50% chance that the two sperms have an identical allele at the same locus, which leads to an identical genotype between endosperm and embryo, and thus HF event cannot be detected even if the HF has occurred (Figure 1). The HF event missed by using one marker can be detected using additional markers. Each additional, independent, marker will detect 50% of the HF events that have been missed by the previous markers. Therefore, as more polymorphic markers are used, the estimated HF frequency becomes closer to the real frequency. The number of markers (*m*) required to have the probability of *p* to detect an HF event can be obtained using *m* = log (1–*p*)/log (1/2). To have a 99% probability of detecting all HF events, we need to use at least seven markers simultaneously. However, the number of HF events that can be identified by each additional marker will decrease drastically. Therefore, we can use one polymorphic SSR marker to detect HF events and then double the number detected to infer the real HF frequency. PCR and SSR genotyping were performed as described in [14].

### Confirmation of hetero-fertilization events using a large number of markers

Four plants from each of two F_2_ populations (HP2 and HP3) identified by SSR markers as derived from hetero-fertilization were genotyped using chip-based SNP markers to confirm the HF events and to testify the probability of detecting HF events using one marker. SNP marker development, genotyping and scoring have been reported elsewhere [20]. SNP genotyping was carried out using an Illumina BeadStation 500 G (Illumina, Inc., San Diego, CA, USA) at the Cornell University Life Sciences Core Laboratories Center using the protocols described in [21]. A total of 5 µL of 50 ng/ µL genomic DNA was used to make single-use DNA as required by the genotyping system which was arranged in Sentrix Array Matrices (SAMs) each with 96 samples. The GoldenGate Assay using a SNP chip containing 1536 markers was then hybridized to the SAMs for genotype analysis. Only 1032 informative and high-quality SNP markers were used in the data analysis.
